# Educational Intervention Increased Referrals to Allopathic Care by Traditional Healers in Three High HIV-Prevalence Rural Districts in Mozambique

**DOI:** 10.1371/journal.pone.0070326

**Published:** 2013-08-01

**Authors:** Carolyn M. Audet, José Salato, Meridith Blevins, David Amsalem, Sten H. Vermund, Felisbela Gaspar

**Affiliations:** 1 Institute for Global Health Vanderbilt University School of Medicine, Nashville, Tennessee, United States of America; 2 Department of Preventive Medicine, Vanderbilt University School of Medicine, Nashville, Tennessee, United States of America; 3 Department of Biostatistics, Vanderbilt University School of Medicine, Nashville, Tennessee, United States of America; 4 Department of Pediatrics, Vanderbilt University School of Medicine, Nashville, Tennessee, United States of America; 5 Friends in Global Health, Quelimane, Mozambique; 6 Ministério da Saúde (Ministry of Health), Maputo, Mozambique; Indiana University and Moi University, United States of America

## Abstract

**Introduction:**

Delayed uptake of clinical services impedes favorable clinical outcomes in Mozambique. Care is delayed among patients who initiate care with traditional healers; patients with conditions like human immunodeficiency virus (HIV) or tuberculosis are rarely referred to the health system in a timely fashion.

**Methods:**

We conducted a pre-post educational intervention with traditional healers, assessing healer referral rates and HIV knowledge in three rural districts in Zambézia Province.

**Results:**

The median monthly referral rate prior to the intervention was 0.25 patients (interquartile range [IQR]: 0–0.54) compared with a post-intervention rate of 0.34 patients (IQR: 0–0.71), a 35% increase (p = 0.046). A median HIV knowledge score of 67% (IQR: 59–78) was noted 4-months pre-intervention and a median score of 81% (IQR: 74–89) was recorded 2½ months post-intervention (p<0.001). One hundred and eleven healers referred 127 adults, 36 pregnant women, and 188 children to health facilities. Referred patients were most likely to be diagnosed with bronchopneumonia (20% adults; 13% children) and/or malaria (15% adults; 37% children). Of 315 non-pregnant persons referred, 3.5% were tested for HIV and 2.5% were tested for tuberculosis.

**Discussion:**

We engaged traditional healers with some success; referral rates were low, but increased post-intervention. Once seen in the clinics, patients were rarely tested for HIV or tuberculosis, though symptoms suggested screening was indicated. We found increased referral rates through an inexpensive intervention with traditional healers, a viable, cost-effective method of directing patients to health facilities. However, quality improvement within the clinics is necessary before a substantial impact can be expected.

## Introduction

Timely testing and engagement into care among those infected with human immunodeficiency virus (HIV), tuberculosis (TB), and malaria and infection remains a large public health challenge in sub-Saharan Africa[1]–[3]. Testing is essential to starting the cascade of medical and behavioural interventions designed to improve a patient’s health and reduce their infectiousness [4]–[7]. For example, persons who test positive for HIV are counselled to reduce their sexual risk behaviours, often resulting in increased condom use [8]. Those successfully initiated on ART have lower viral loads [9], [10], resulting in lower rates of transmission to their sexual partners [11]–[14], and if pregnant, are less likely to pass the virus to their infants [15], [16]. Of course, early TB detection and treatment reduces TB infectiousness [17] and improves patient outcomes [18]. An early malaria diagnosis typically leads to reduced morbidity and mortality [19], [20]. Despite the availability of clinical services, slow uptake and/or delayed help seeking behaviour remains a barrier to improving patient outcomes for many diseases in low and middle income nations (LMICs) [21]–[26].

In sub-Saharan Africa people commonly seek initial health consultations from a traditional or religious healer [27]–[31]. Symptoms, perceived source of the illness, or failed treatment from traditional healers can all motivate a patient to seek clinical services [27], [28]. People visit traditional healers due to social acceptability, perceived source of illness, confidence in healer treatment (and/or a lack of confidence in the health center), easy access, low cost, as well as the perceived fit of a healer’s explanation of illness with expectations of the local culture [32]–[35]. Physicians often openly disapprove of patients who first see a healer for treatment, often resulting in animosity between healers and clinicians, as well as between scolded patients and their clinicians [28], [36], [37].

Given the level of trust in the community and the absolute numbers of healers, the incorporation of traditional healers into the formal health system could help increase early diagnosis and therapy with prompt referrals [31], [38]–[43]. Efforts to increase healer knowledge about specific diseases, improve relationships with allopathic practitioners, and reduce delays to allopathic care have been piloted with traditional healers in diverse countries, including Brazil, Uganda, Kenya, Ghana, Cameroon, Lesotho, Gambia, Nepal, and India [24], [44]–[58]. While educational and motivational interventions designed to change healer behavior are common, formal assessment of their effect on patient referral or patient health outcomes is limited [46], [50], [57], [59]. In Mozambique, both the Ministry of Health and traditional healers are enthusiastic about the creation of a formal partnership [37]. To this end, the Ministério da Saúde (MISAU; Mozambican Ministry of Health), along with several formal healer organizations in Zambézia province, and the Vanderbilt University affiliated non-governmental organization Friends in Global Health (FGH) collaborated to design a formal system for documenting healer referrals to the health facility and for providing feedback to healers regarding patient diagnoses.

## Methods

### Study Locations

**Figure 1:**
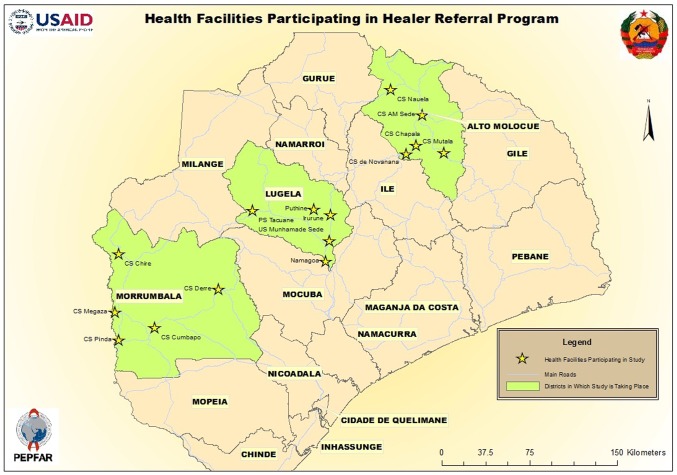
Map study districts in Zambézia Province (Friends in Global Health SCIP project supported by MISAU and USAID).

This study was conducted from February-September 2012, in 15 health facilities located in the districts of Morrumbala, Alto Molócuè, and Lugela in Zambézia Province, Mozambique (Figure 1). We were assisted by the organization for traditional healers, the *Associação dos Médicos Tradicionais de Moçambique* (AMETRAMO).

### Participant Eligibility and Recruitment

Traditional healers were eligible to participate if they were an adult (≥18 years), lived and practiced traditional medicine in the catchment area (≤10 km) surrounding a target health facility (either the district level hospital or any of four small community-level health posts), a member of the AMETRAMO organization, and capable of providing informed consent. A convenience sample of healers was recruited from healers who were members of AMETRAMO in each region. Healers were informed about the study by one of two of us (CMA and JS) during monthly AMETRAMO meetings in January–February 2012. Healers were recruited if they met the eligibility requirements, were willing to use a referral form to identify their referrals to the health facility, and agreed to participate in a health training session for three days at the midpoint of the study. Healers who were interested learning more about the study met with study personnel in a private room following the meeting. Once the healer agreed to participate, they completed the consent form and the baseline interviewer-administered knowledge questionnaire. Healers were enrolled on a “first come, first enrolled” basis until we recruited an average of 37 participants in each district until sample size requirements were met. The questionnaire took approximately 30–60 minutes to complete; no financial incentive was provided. The same tool was administered a second time to the same participants in September 2012. Study data were collected and managed using REDCap™ electronic data management software [60].

### Intervention

**Figure 2:**
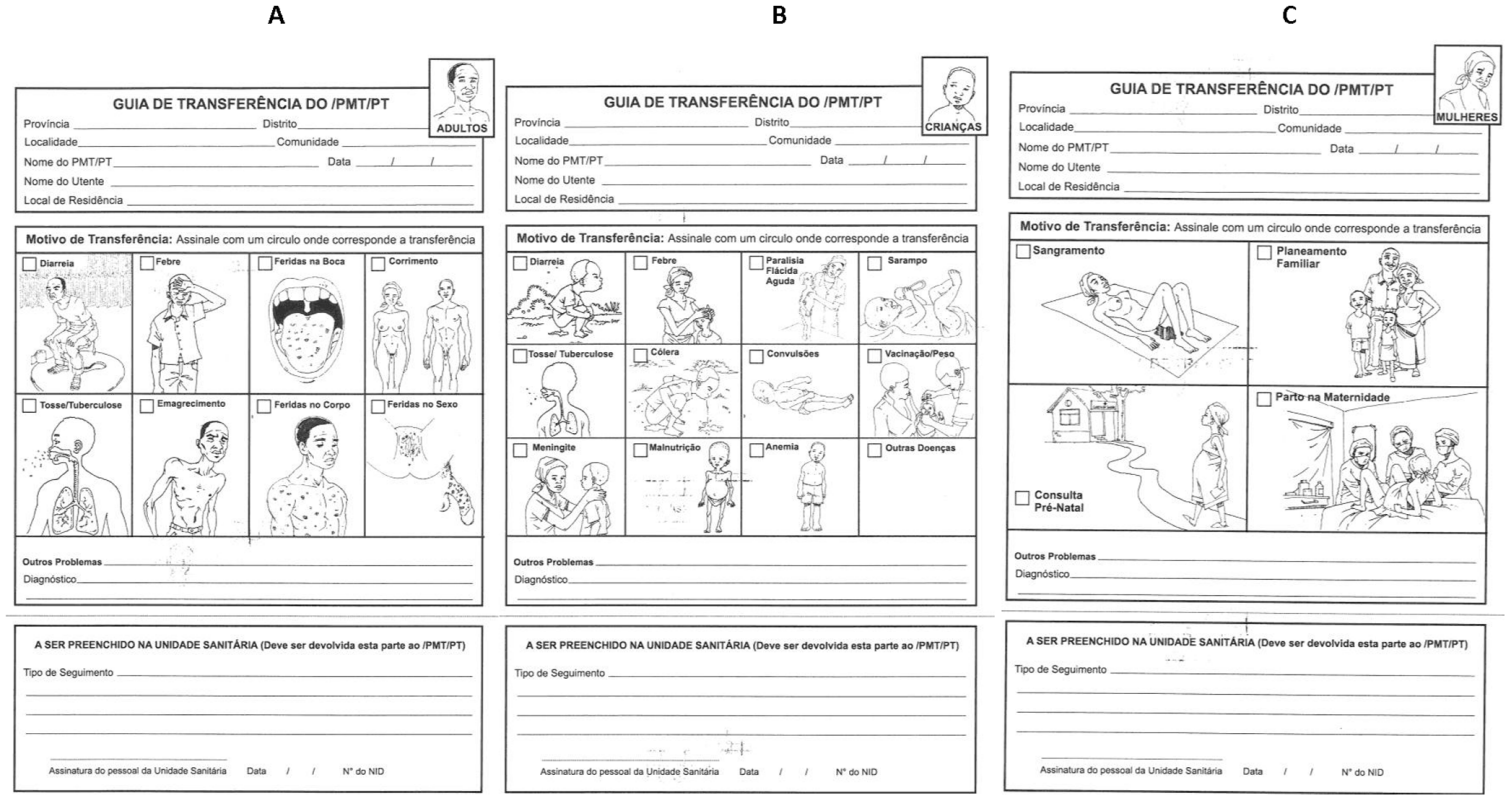
Adult (A), Child (B), and Pregnant Woman (C) Referral Form.

Following the completion of the first questionnaire, healers were provided a two hour training session on how to use a new referral form designed to track healer referrals to the health system (Figure 2). At this baseline data collection time period, healers were provided only minimal direction about whom to refer, but were instructed carefully how to use the pictorial form to document their referral. Each healer was given a packet of 120 forms (40 each for adults, pregnant women, and children) with instructions on how to secure additional forms from our collaborators in the communities. Healers were asked to refer patients to the health facility as they had in the past (either direct accompaniment or by providing the form to the patient directly and giving them instructions to visit the clinic). We followed up with healers to assess any difficulties with filling out the forms and with clinical sites to collect the referral forms once each month. Based on the number of incomplete referral forms collected at the health facilities in the first few months, two additional training sessions with healers were conducted in each district to improve completion. A binder for referral form collection was placed in the patient intake (or consult) room(s) at each clinic or hospital for the collection of referral forms. Physician, nurses, and medical technicians, were asked to file referral forms as they were collected at the hospital.

In June–July 2012, FGH implemented a primary health training course for healers in each study district capital. The training course was designed to help healers identify patients with HIV, TB, malaria, malnutrition, mental illness, and diarrhea and provide information about the importance of early detection and referral for standard methods of treatment. Training in disease identification and in the use of referral forms was conducted bilingually (in Portuguese as well as in the relevant local languages of Elowme, Sena, or Manhaua). All training related costs, including travel and meals were covered by the project; however, no additional financial incentives were provided to the healers. Two and one half months after the intervention, we retested healer HIV knowledge through interviewer-administered knowledge questionnaires, identical to the baseline, in their home communities.

### Measures

#### The questionnaire

The questionnaire that was administered twice, pre-training and post-training, to each healer collected information on demographics, Portuguese proficiency, general knowledge about HIV-related knowledge, tuberculosis, malaria, nutrition, and diarrhea, and using the previously validated HIV KQ-27 knowledge scale [61].

#### Referral forms

Referral forms were designed through collaboration with FGH personnel and the investigators, MISAU’s Instituto Nacional de Medicina Tradicional (National Institute of Traditional Medicine within the Ministry of Health), and the Zambézia branch of AMETRAMO. Referral forms include the date of patient referral, provide the name of the referring healer, and include information about the patient symptoms and diagnosis. These referral forms were collected from each health facility once per month throughout the study period.

### Analysis

The monthly rate of patient referral for each healer is calculated as the number of patients referred prior to and after intervention divided by the duration in months of pre- and post-intervention. We compared pre- and post-monthly rate of referral using a paired Wilcoxon rank sum test. To estimate which healer characteristics were associated with better uptake of referral practices we used multivariable linear regression. The model included age, education, sex, district, Portuguese speaking ability, and post-intervention HIV score. We describe those healers with no referrals to those with ≥1 referrals during the study intervention. To compare the distribution of healer characteristics by any referral, we employ chi-square and Wilcoxon rank sum tests. From referral forms, we describe patient location, symptoms, clinical diagnosis, and HIV status by referral pre- and post-intervention. R-software 2.15.1 (www.r-project.org) was used for data analyses. The study was reviewed and approved after iterations by the Vanderbilt Institutional Review Board and the Comité Nacional de Bioética a Saúde in Mozambique. All participants provided written consent to participate in the study; this procedure was approved by both ethics committees.

## Results

We enrolled 111 healers in the study. We presented the study to 188 traditional healers at 15 AMETRAMO meetings. Interest was indicated from 147 (78.2%), of whom 111 were enrolled in the study. All of the 147 healers who spoke with a study coordinator agreed to participate; we had to turn away 36 potential participants after the study was filled. Healers had a median age of 51 years (IQR: 46–61), 60% were men, and had obtained a median of one year of formal education (IQR: 0–4). Healers reported a low level of oral Portuguese proficiency: 33% were fluent, 25% spoke some Portuguese, and 42% had no Portuguese speaking ability. Seventy-six percent of the healers in the study had referred a patient to the health facility in the past, with no statistically significant differences in overall referral frequencies among healers in different communities (Table 1).

**Table 1:** Characteristics of 111 Traditional Healers from three rural districts in Zambézia Province, Mozambique, comparing those who referred to allopathic clinics with those who did not refer.

	No referrals	Any referral(s)	All	P-value^b^
	(n = 27)	(n = 84)	(n = 111)	
District, n (%)				0.35
Alto Molócuè	11 (41%)	22 (26%)	33 (30%)	
Lugela	9 (33%)	33 (39%)	42 (38%)	
Morrumbala	7 (26%)	29 (35%)	36 (32%)	
Age, Median (Interquartile range)^a^	50 (48–61)	52 (45–61)	51 (46–61)	0.36
* Missing, n (%)*	*1 (4%)*	*5 (6%)*	*6 (5%)*	
Speaks Portuguese, n (%)				0.95
No	12 (44%)	33 (42%)	45 (42%)	
A little	6 (22%)	20 (25%)	26 (25%)	
Yes	9 (33%)	26 (33%)	35 (33%)	
Sex, n (%)				0.32
Female	8 (30%)	34 (43%)	42 (40%)	
Male	19 (70%)	45 (57%)	64 (60%)	
Distance (time)^a^	40 (5–60)	33 (24–60)	35 (18–60)	0.58
* Missing, n (%)*	*14 (52%)*	*38 (45%)*	*52 (47%)*	
Distance (km)^a^	2 (1–4)	2 (1–5)	2 (1–5)	0.87
* Missing, n(%)*	*14 (52%)*	*47 (56%)*	*61 (55%)*	
Education in years^a^	1 (0–3)	1 (0–4)	1 (0–3)	0.98
Pre-Intervention HIV Knowledge Score^a^	63 (56–71)	70 (63–78)	67 (59–78)	0.032
Post-Intervention HIV Knowledge Score^a^	81 (74–88)	81 (73–90)	81 (74–89)	0.56
* Missing, n(%)*	*5 (19%)*	*16 (19%)*	*21 (19%)*	
Mean difference in Pre- and Post-Intervention HIV Knowledge Scores^a^	15 (3–21)	10 (0–19)	11 (1–19)	0.29
* Missing, n(%)*	*5 (19%)*	*16 (19%)*	*21 (19%)*	

a Continuous data are summarized as median (interquartile range).

b To compare the distribution of study characteristics for healers with any referral, we employ chi-square tests. Similarly, we use a Wilcoxon rank sum test for continuous variables by any referral.

During the study period, healers referred 351 persons –127 adults, 36 pregnant women, and 188 children – to the health facility for treatment. Among adults referred, the most common symptoms were (more than one symptom could be named): fever (37%), cough (24%), and diarrhea (13%) (Table 2). Pediatric patients were referred for fever (42%), cough (15%), and convulsions (9%) (Table 3), while pregnant women were referred for delivery (37%) and prenatal care (24%) (data not shown). In addition to information about the symptoms that may have spurred healers to refer, we also gathered data about patient diagnoses by the allopathic clinicians. Referred adults and children were most likely to have bronchopneumonia (20% of adults; 13% of children) and malaria (15% of adults; 37% of children) diagnosed syndromically. Excluding pregnant women who are nearly all tested on an opt-out basis, only 11 of 315 (3.5%) referred adults and children were tested for HIV, with 6 of 11 (54%) testing positive. Again excluding pregnant women, eight patients (2.5%) were tested for TB, with 3 of 8 (38%) testing positive.

**Table 2:** Summary of adult referrals from 52 of 111 Traditional Healers from three rural districts in Zambézia Province, Mozambique, comparing referrals occurring Pre- and Post-Intervention.

	Pre-intervention referrals	Post-intervention referrals	Both pre−/post-intervention referrals
	(n = 69)	(n = 58)	(n = 127)
District, n(%)			
Alto Molócuè	21 (30%)	11 (19%)	32 (25%)
Lugela	36 (52%)	28 (48%)	64 (50%)
Morrumbala	12 (17%)	19 (33%)	31 (24%)
Symptoms with referral, n(%)^a^			
Diarrhea	10 (14%)	7 (12%)	17 (13%)
Fever	25 (36%)	22 (38%)	47 (37%)
Spots on Tongue	1 (1%)	2 (3%)	3 (2%)
Discharge from genitals	9 (13%)	6 (10%)	15 (12%)
Cough (possible tuberculosis)	15 (22%)	15 (26%)	30 (24%)
Emaciation	3 (4%)	4 (7%)	7 (6%)
Body sores	4 (6%)	5 (9%)	9 (7%)
Spots on genitals	3 (4%)	5 (9%)	8 (6%)
Other problems	0 (0%)	2 (3%)	2 (2%)
Diagnosis at clinic, n(%)^a^			
Bronchopulmonary pneumonia	12 (17%)	13 (22%)	25 (20%)
Malaria	12 (17%)	7 (12%)	19 (15%)
Gastrointestinal problem	4 (6%)	2 (3%)	6 (5%)
Sexually transmitted infection	5 (7%)	3 (5%)	8 (6%)
HIV infection	2 (3%)	2 (3%)	4 (3%)
Tuberculosis	5 (7%)	2 (3%)	7 (6%)
Emergency	1 (1%)	2 (3%)	3 (2%)
Other^b^	18 (26%)	15 (26%)	33 (26%)

a Percentages may sum to more than 100% given multiple conditions.

b e.g. skin infection, broken bone, vertigo, snake bite.

**Table 3:** Summary of child referrals from 64 of the 111 Traditional Healers from three rural districts in Zambézia Province, Mozambique, comparing referrals occurring Pre- and Post-Intervention.

	Pre-intervention referrals	Post-intervention referrals	Both pre−/post-intervention referrals
	(n = 93)	(n = 95)	(n = 188)
District, n (%)			
Alto Molócuè	33 (35%)	26 (27%)	59 (31%)
Lugela	33 (35%)	31 (33%)	64 (34%)
Morrumbala	27 (29%)	38 (40%)	65 (35%)
Symptoms/Referral, n (%)^a^			
Diarrhea	10 (11%)	7 (7%)	17 (9%)
Fever	42 (45%)	37 (39%)	79 (42%)
“Flaccid paralysis”	6 (6%)	4 (4%)	10 (5%)
Measles	1 (1%)	5 (5%)	6 (3%)
Cough associated with TB	17 (18%)	12 (13%)	29 (15%)
Cholera	8 (9%)	5 (5%)	13 (7%)
Convulsions	8 (9%)	10 (11%)	18 (10%)
Anemia	7 (8%)	3 (3%)	10 (5%)
Other problems	7 (8%)	14 (15%)	21 (11%)
Diagnosis, n (%)^a^			
Bronchopulmonary pneumonia	8 (9%)	16 (17%)	24 (13%)
Malaria	37 (40%)	32 (34%)	69 (37%)
Gastrointestinal disorder	11 (12%)	10 (11%)	21 (11%)
Schistosomaisis	4 (4%)	6 (6%)	10 (5%)
HIV infection	1 (1%)	0 (0%)	1 (1%)
Respiratory disorder	0 (0%)	2 (2%)	2 (1%)
Vaccination	1 (1%)	0 (0%)	1 (1%)
Anemia	1 (1%)	0 (0%)	1 (1%)
Other^b^	8 (9%)	22 (23%)	30 (16%)

a Percentages may sum to more than 100% given multiple conditions.

b e.g. leprosy, ear infection, oral thrush.

HIV knowledge (measured 4 months pre-intervention and 2.5 months post-intervention) increased significantly. Healers had a median pre-intervention HIV knowledge score of 67% (IQR: 59–78) and a median post-intervention score of 81% (IQR: 74–89; p<0.001). Overall, healers improved their scores on 26 of the 27 questions. Key areas where knowledge increased included: (1) a blood test for HIV exists (77% pre vs. 98% post); (2) antiretroviral therapy (ART) can improve the health of people living with HIV (65% vs. 89%); and (3) healers cannot provide herbal or spells to protect patients from HIV (59% vs. 81%). Misunderstandings about transmission via mosquitoes (43% vs. 63%), utensils (59% vs. 79%), or coughing/sneezing (58% vs. 76%) were resolved in the minds of more healers post-intervention. The only question in which healers had lower knowledge post-intervention highlighted the belief that eating healthy foods could protect a person from getting HIV (60% vs. 54%).

The number of referrals increased significantly post-intervention. The median monthly referral rate prior to the intervention was 0.25 patients (IQR: 0–0.54) compared with a post-intervention rate of 0.34 patients (IQR: 0–0.71; p = 0.046); this amounts to a 35% increase overall. In multivariable linear regression, no associations were noted among healers who had increasing uptake of referral practices, including improved HIV knowledge (p = 0.50), Portuguese speaking ability (p = 0.63), or age of healer (p = 0.66). In fact, model fit was very poor (R^2^ = 0.055), suggesting that healer characteristics included in the model poorly predicted the change in referral rate (Table 4).

**Table 4:** Multivariable model effects for change in referrals pre- to post-intervention from 84 Traditional Healers who referred at least one person from three rural districts in Zambézia Province, Mozambique by Healer characteristics and post-intervention HIV knowledge.

	Estimate (95% CI)	P-value
Age (per 10 years)	0.022 (−0.11, 0.16)	0.75
Education (per 1 year)	0.034 (−0.043, 0.11)	0.39
Female	0.002 (−0.34, 0.35)	0.99
District		0.27
Lugela (ref)	0	
Alto Molócuè	−0.20 (−0.61, 0.21)	
Morrumbala	0.16 (−0.25, 0.57)	
Portuguese speaking ability		0.63
None (ref)	0	
A little	−0.064 (−0.49, 0.37)	
Fluent	−0.21 (−0.66, 0.23)	
Post-intervention HIV knowledge (per 15 points)	0.11 (−0.13, 0.34)	0.37

Referral forms were generally filled out well by traditional healers, accuracy appeared to improve over time. Only 19 (5%) of forms were missing the name of the traditional healer, 38 (11%) were missing the date of referral, and 351 (100%) referral forms included data about the nature of the patient symptoms (either by circling pictures depicting symptoms or writing additional information on the forms). Clinical data were included with less regularity, with 22% of the forms lacking a diagnosis.

## Discussion

Our educational intervention resulted in a 35% increase in healer referral rates, though the monthly frequencies of referral remained low. Fever, cough, and diarrhea were the most common symptoms among referred patients in both the pre- and post-intervention period. Similarly, the reasons for referring pregnant women were unchanged pre- and post-intervention; women were referred equally for prenatal care services or delivery, with a smaller number referred for treatment of vaginal bleeding. That low-cost engagement of traditional healers can foster such referrals is encouraging.

We were surprised, however, that under 5% of referred patients were tested for HIV and/or TB. Based on the high rates of HIV and TB in these districts, [62] coupled with the large number of patients referred for cough, emaciation, fever, white spittle, and/or skin lesions, we expected a large number of patients to be tested for both infections. The failure to routinely test patients for HIV at all 15 clinics suggests a systemic problem that needs to be addressed and may explain why patients are continuing to be first tested only after they suffer symptoms of advanced HIV disease [63]. There were several stock outs of HIV testing kits during our study (CMA and JS, personal observations); nonetheless, clinicians were frank with us that routine testing has not become integrated into clinical practice.

As with other studies measuring HIV knowledge change in traditional healers after an educational training [29], [48], [52], [54], [59], [64] we found a sustained increase in HIV knowledge among healers. While the three-day educational intervention was associated with increasing HIV knowledge by 14%, it is interesting to note that we failed to detect an association between this increase and change in referral practices. This is similar to the finding from a South African study [59]. We do not yet know, then, what motivated some healers to increase their referrals. We hypothesize that increased contact with healers (by our team and with the health facilities) created a greater feeling of community and inclusiveness by healers. We believe this relationship building helped to decrease feelings of hostility between the healers and the clinic health care providers. In follow-up with both healers and clinicians, we found that some continued to collaborate in ways we originally had not envisioned. For example, clinicians in Morrumbala established monthly meetings with healers to discuss referred patients. In Lugela, traditional healers came to the district hospital to follow up on their patients and to ensure clinicians were keeping their referral forms. We are now documenting these new activities and the impact of increased contact between the system and healers on healer attitudes and behavior.

The acceptability of our novel referral form and healers ability to fill out the form accurately surpassed our expectations, with 100% completion rates for the “symptom” section, 95% for the date of referral, and 89% for their own names. Patient and healer names were sometimes reversed (healer name where the patient name should be written and vice versa) suggesting some confusion and difficulty in reading the Portuguese forms. In the future, we will consider adding pictures to this section to improve comprehension. The clinical diagnosis section was only completed 78% of the time. We did not provide clinicians any incentive to complete the form and believe that the improvement of the absolute numbers of referrals and quality of the referral form represent future implementation science challenges.

Study strengths included the highly acceptable use of referral forms among healers living in three rural districts, and generalizability of healer referrals for rural catchment areas varying in size and distance from hospitals or health posts in all three districts (data not shown). This suggests the acceptability of the referral forms and the scalability of the project in a variety of rural settings if deployed elsewhere in Mozambique. The high rate of form completion from healers suggested that pictorial forms (Figure 2) can be used even when healers have low rates of literacy. The study also highlighted several challenges. Healers complained that clinicians did not always file the referral form presented by the patient, throwing some in the garbage. This was supported by our discovery of several diagnosis sections (the lower section of the referral forms filled out by the clinician) that had been returned to healers without their matching symptom section filed at the clinic. Clinic personnel with whom we spoke suggested that the nurses on duty during the evenings and weekends were less likely to file the forms, although we cannot confirm this. Study weaknesses include the convenience sample recruited in a pre-post design employed to make this study feasible. The pre-post study design does not control for calendar effect. The use of a convenience sample of healers’ further limits our conclusions as self-selection of healers interested in participating may better uptake the intervention. The possibility that some forms were not saved would mean that the study would undercount healer referrals; however, cross-checking the duplicate healer records with those at the health facilities, we estimate the number of discarded referral forms to be <10%. Because we lacked information on the patients who were referred by healers but did not go to the clinic and those patients who were seen by healers and not referred to clinic, we cannot assess the actual rate of referral and the effectiveness of healer referral, but only the rate of successful linkage. A further limitation of this study is future sustainability in the context of an under-capacitated Ministry of Health; despite purposeful efforts not to monetize healer involvement, any referral system would be limited by their ability to engage healers and clinicians, and to ensure appropriate distribution of referral forms.

This study demonstrates the feasibility of implementing a referral system with healers in Mozambique. Our ability to increase referral rates through an inexpensive intervention suggests a formal relationship with traditional healers may be a viable, cost-effective method of directing patients suffering from acute or chronic illness to the health facility. Whether electronic mechanisms of referral, such as mobile phone short messaging systems, might be deployed is unknown in this rural setting. The possibility of engaging healers in additional clinical tasks, including community-based HIV testing is a possibility, but would require additional support from the Ministry of Health. Given the large number of healers living in rural and remote areas with limited access to formal health services, traditional healers can be pivotal in the recognition and initiation of referral for timely testing for illness such as HIV and TB, assuming clinicians can be persuaded to implement more routine testing. Our study suggests that with minimal cost, healers and clinicians can collaborate to improve patient care.

## References

[1] Raviglione MC (2007) The new Stop TB Strategy and the Global Plan to Stop TB, 2006–2015. Bull World Health Organ 85: 327. 17639210 10.2471/06.038513 PMC2636638

[2] Joint United Nations Programme on HIV/AIDS (UNAIDS) (2008) Report on the golbal HIV/AIDS epidemic.

[3] Hopkins H, Asiimwe C, Bell D (2009) Access to antimalarial therapy: accurate diagnosis is essential to achieving long term goals. BMJ 339: b2606. 19584113 10.1136/bmj.b2606

[4] Hayes R, Sabapathy K, Fidler S (2011) Universal testing and treatment as an HIV prevention strategy: research questions and methods. Curr HIV Res 9: 429–445. 21999778 10.2174/157016211798038515 PMC3520051

[5] Zachariah R, Harries AD, Philips M, Arnould L, Sabapathy K, et al (2010) Antiretroviral therapy for HIV prevention: many concerns and challenges, but are there ways forward in sub-Saharan Africa? Trans R Soc Trop Med Hyg 104: 387–391. 20116814 10.1016/j.trstmh.2010.01.004

[6] Hensen B, Baggaley R, Wong VJ, Grabbe KL, Shaffer N, et al (2012) Universal voluntary HIV testing in antenatal care settings: a review of the contribution of provider-initiated testing & counselling. Trop Med Int Health 17: 59–70. 22032300 10.1111/j.1365-3156.2011.02893.x

[7] Bassett IV, Walensky RP (2010) Integrating HIV screening into routine health care in resource-limited settings. Clin Infect Dis 50 Suppl 3S77–84. 20397960 10.1086/651477 PMC3515844

[8] Bunnell R, Ekwaru JP, Solberg P, Wamai N, Bikaako-Kajura W, et al (2006) Changes in sexual behavior and risk of HIV transmission after antiretroviral therapy and prevention interventions in rural Uganda. AIDS 20: 85–92. 16327323 10.1097/01.aids.0000196566.40702.28

[9] Vernazza PL, Gilliam BL, Flepp M, Dyer JR, Frank AC, et al (1997) Effect of antiviral treatment on the shedding of HIV-1 in semen. AIDS 11: 1249–1254. 9256943 10.1097/00002030-199710000-00008

[10] Cu-Uvin S, Caliendo AM, Reinert S, Chang A, Juliano-Remollino C, et al (2000) Effect of highly active antiretroviral therapy on cervicovaginal HIV-1 RNA. AIDS 14: 415–421. 10770544 10.1097/00002030-200003100-00015

[11] Quinn TC, Wawer MJ, Sewankambo N, Serwadda D, Li C, et al (2000) Viral load and heterosexual transmission of human immunodeficiency virus type 1. Rakai Project Study Group. N Engl J Med 342: 921–929. 10738050 10.1056/NEJM200003303421303

[12] Anglemyer A, Rutherford GW, Egger M, Siegfried N (2011) Antiretroviral therapy for prevention of HIV transmission in HIV-discordant couples. Cochrane Database Syst Rev 5: CD009153. 10.1002/14651858.CD009153 21563172

[13] Fideli US, Allen SA, Musonda R, Trask S, Hahn BH, et al (2001) Virologic and immunologic determinants of heterosexual transmission of human immunodeficiency virus type 1 in Africa. AIDS Res Hum Retroviruses 17: 901–910. 11461676 10.1089/088922201750290023 PMC2748905

[14] Cohen MS, Chen YQ, McCauley M, Gamble T, Hosseinipour MC, et al (2011) Prevention of HIV-1 infection with early antiretroviral therapy. N Engl J Med 365: 493–505. 21767103 10.1056/NEJMoa1105243 PMC3200068

[15] Brocklehurst P, Volmink J (2002) Antiretrovirals for reducing the risk of mother-to-child transmission of HIV infection. Cochrane Database Syst Rev: CD003510. 10.1002/14651858.CD003510 11869666

[16] Volmink J, Siegfried NL, van der Merwe L, Brocklehurst P (2007) Antiretrovirals for reducing the risk of mother-to-child transmission of HIV infection. Cochrane Database Syst Rev: CD003510. 10.1002/14651858.CD003510.pub2 17253490

[17] Fitzwater SP, Caviedes L, Gilman RH, Coronel J, LaChira D, et al (2010) Prolonged infectiousness of tuberculosis patients in a directly observed therapy short-course program with standardized therapy. Clin Infect Dis 51: 371–378. 20624064 10.1086/655127 PMC4465448

[18] World Health Organization (2010) Treatment of Tuberculosis Guidelines: Forth Edition. Geneva, Switzerland: WHO Press.

[19] Bell D, Wongsrichanalai C, Barnwell JW (2006) Ensuring quality and access for malaria diagnosis: how can it be achieved? Nat Rev Microbiol 4: S7–20. 10.1038/nrmicro1525 17003770

[20] World Health Organization (2012) Malaria: Diagnosis and Treatment. Geneva, Switerzland: WHO press.

[21] Ahorlu CK, Koram KA, Ahorlu C, de Savigny D, Weiss MG (2006) Socio-cultural determinants of treatment delay for childhood malaria in southern Ghana. Trop Med Int Health 11: 1022–1031. 16827703 10.1111/j.1365-3156.2006.01660.x

[22] van Dyk AC, van Dyk PJ (2003) "To know or not to know": service-related barriers to voluntary HIV counseling and testing (VCT) in South Africa. Curationis 26: 4–10. 10.4102/curationis.v26i1.1289 14509113

[23] Matovu JK, Makumbi FE (2007) Expanding access to voluntary HIV counselling and testing in sub-Saharan Africa: alternative approaches for improving uptake, 2001–2007. Trop Med Int Health 12: 1315–1322. 17949401 10.1111/j.1365-3156.2007.01923.x

[24] Bwambale F, Ssali S, Byaruhanga S, Kalyango J, Karamagi C (2008) Voluntary HIV counselling and testing among men in rural western Uganda: Implications for HIV prevention. BMC Public Health 8: 263. 18664301 10.1186/1471-2458-8-263 PMC2529297

[25] Getahun A, Deribe K, Deribew A (2010) Determinants of delay in malaria treatment-seeking behaviour for under-five children in south-west Ethiopia: a case control study. Malar J 9: 320. 21070644 10.1186/1475-2875-9-320 PMC2988828

[26] Njau IW, Karanja SM, Wanzala P, Omolo JO (2012) Factors associated with late presentation of suspected tuberculosis cases to tuberculosis management facilities: The case in Dagoretti district, Nairobi, Kenya. Pan Afr Med J 12: 93. 23077714 PMC3473979

[27] Audet CM, Blevins M, Moon TD, Vergara AE, Vermund SH, et al (2012) Health Seeking Behavior in Zambezia Province, Mozambique. SAHARAJ 9: 41–46. 10.1080/17290376.2012.665257 PMC3868210 23237020

[28] Audet C, Blevins M, Rosenberg C, Farnsworth S, Fernandez J, et al.. (2012) Traditional healer consultation is associated with a 3 month delay in HIV testing among symptomatic HIV-positive patients in rural Mozambique. XIX International AIDS Conference Washington, CD.

[29] Green EC, Zokwe B, Dupree JD (1995) The experience of an AIDS prevention program focused on South African traditional healers. Soc Sci Med 40: 503–515. 7725124 10.1016/0277-9536(94)e0105-2

[30] (1998) Poverty, sexual and reproductive health: recommendations for family planning associations. Sex Health Exch: 8. 12294689

[31] King R (2000) Collaboration with traditional healers in HIV/AIDS prevention and care in sub-Saharan Africa: a literature review.

[32] Babb DA, Pemba L, Seatlanyane P, Charalambous S, Churchyard GJ, et al (2007) Use of traditional medicine by HIV-infected individuals in South Africa in the era of antiretroviral therapy. Psychol Health Med 12: 314–320. 17510901 10.1080/13548500600621511

[33] Green EC (1999) Traditional healers and AIDS in Uganda. Journal of Alternative and Complementary Medince 6: 1–2. 10.1089/acm.2000.6.1 10706229

[34] Pinkoane MG, Greeff M, Williams MJ (2005) The patient relationship and therapeutic techniques of the South Sotho traditional healer. Curationis 28: 20–30. 10.4102/curationis.v28i4.1005 16450556

[35] Stekelenburg J, Jager BE, Kolk PR, Westen EH, van der Kwaak A, et al (2005) Health care seeking behaviour and utilisation of traditional healers in Kalabo, Zambia. Health Policy 71: 67–81. 15563994 10.1016/j.healthpol.2004.05.008

[36] Groh KE, Audet CM, Tonela A, Sidat M, Vergara AE, et al.. (2011) Barriers to antiretroviral therapy adherence in rural Mozambique. BMC Public Health 11. 10.1186/1471-2458-11-650 PMC3171727 21846344

[37] Audet CM, Blevins M, Moon TD, M S, Shepherd BE, et al (2012) HIV/AIDS-Related Attitudes and Practices Among Traditional Healers in Zambezia Province, Mozambique. Journal of Alternative and Complementary Medince 18: 1–9. 10.1089/acm.2011.0682 PMC3513988 23171035

[38] Burnett A, Baggaley R, Ndovi-MacMillan M, Sulwe J, Hang'omba B, et al (1999) Caring for people with HIV in Zambia: are traditional healers and formal health workers willing to work together? AIDS Care 11: 481–491. 10533542 10.1080/09540129947875

[39] Fleming J (1995) Mozambican healers join government in fight against AIDS. J Int Assoc Physicians AIDS Care 1: 32. 11362312

[40] Kayombo EJ, Uiso FC, Mbwambo ZH, Mahunnah RL, Moshi MJ, et al (2007) Experience of initiating collaboration of traditional healers in managing HIV and AIDS in Tanzania. J Ethnobiol Ethnomed 3: 6. 17257409 10.1186/1746-4269-3-6 PMC1797004

[41] Liverpool J, Alexander R, Johnson M, Ebba EK, Francis S, et al (2004) Western medicine and traditional healers: partners in the fight against HIV/AIDS. J Natl Med Assoc 96: 822–825. 15233493 PMC2568353

[42] Madamombe I (2006) Traditional healers boost primary helath care: Reaching patients missed by modern medicine. African Renewal 19: 10.

[43] PlusNews (2010) South Africa: Traditional healers extend healthcare. IRIN: humanitarian news and analysis. Durban: UN Office for the Coordination of Humanitarian Affairs.

[44] McMillen H (2004) The adapting healer: pioneering through shifting epidemiological and sociocultural landscapes. Soc Sci Med 59: 889–902. 15186892 10.1016/j.socscimed.2003.12.008

[45] Rudolph MJ, Ogunbodede EO, Mistry M (2007) Management of the oral manifestations of HIV/AIDS by traditional healers and care givers. Curationis 30: 56–61. 17515317 10.4102/curationis.v30i1.1051

[46] Colvin M, Gumede L, Grimwade K, Maher D, Wilkinson D (2003) Contribution of traditional healers to a rural tuberculosis control programme in Hlabisa, South Africa. Int J Tuberc Lung Dis 7: S86–91. 12971659

[47] Green EC (1994) AIDS and STDs in Africa: bridging the gap between traditional healing and modern medicine. Boulder: Westview Press.

[48] Poudel KC, Jimba M, Joshi AB, Poudel-Tandukar K, Sharma M, et al (2005) Retention and effectiveness of HIV/AIDS training of traditional healers in far western Nepal. Trop Med Int Health 10: 640–646. 15960702 10.1111/j.1365-3156.2005.01443.x

[49] Furin J (2011) The role of traditional healers in community-based HIV care in rural Lesotho. J Community Health 36: 849–856. 21374087 10.1007/s10900-011-9385-3

[50] Kambo IP, Gupta RN, Kundu AS, Dhillon BS, Saxena HM, et al (1994) Use of traditional medical practitioners to delivery family planning services in Uttar Pradesh. Stud Fam Plann 25: 32–40. 8209393

[51] Harper ME, Hill PC, Bah AH, Manneh K, McAdam KP, et al (2004) Traditional healers participate in tuberculosis control in The Gambia. Int J Tuberc Lung Dis 8: 1266–1268. 15527161

[52] Nations MK, De Souza MA (1997) Umbanda healers as effective AIDS educators: Case-control study in Brazilian urban slums (favelas). Tropical Doctor 27: 60–66. 9204729 10.1177/00494755970270S118

[53] Mbeh GN, Edwards R, Ngufor G, Assah F, Fezeu L, et al (2010) Traditional healers and diabetes: results from a pilot project to train traditional healers to provide health education and appropriate health care practices for diabetes patients in Cameroon. Glob Health Promot 17: 17–26. 10.1177/1757975910363925 20595335

[54] Somse P, Chapko MK, Wata JB, Bondha P, Gonda B, et al (1998) Evaluation of an AIDS training program for traditional healers in the Central African Republic. AIDS Educ Prev 10: 558–564. 9883290

[55] Warren DM, Bova GS, Tregoning MA, Kliewer M (1982) Ghanaian national policy toward indigenous healers. The case of the primary health training for indigenous healers (PRHETIH) program. Soc Sci Med 16: 1873–1881. 7178933 10.1016/0277-9536(82)90448-8

[56] Berger RA, Porter L, Mekisini G, Courtright P (1994) Traditional healers in AIDS control. AIDS 8: 1511–1512. 7818832 10.1097/00002030-199410000-00028

[57] Oswald IH (1983) Are traditional healers the solution to the failures of primary health care in rural Nepal? Soc Sci Med 17: 255–257. 6857288 10.1016/0277-9536(83)90327-1

[58] Ssali A, Butler LM, Kabatesi D, King R, Namugenyi A, et al (2005) Traditional healers for HIV/AIDS prevention and family planning, Kiboga District, Uganda: evaluation of a program to improve practices. AIDS Behav 9: 485–493. 16249945 10.1007/s10461-005-9019-9

[59] Peltzer K, Mngqundaniso N, Petros G (2006) A controlled study of an HIV/AIDS/STI/TB intervention with traditional healers in KwaZulu-Natal, South Africa. AIDS Behav 10: 683–690. 16715347 10.1007/s10461-006-9110-x

[60] Harris PA, Taylor R, Thielke R, Payne J, Gonzalez N, et al (2009) Research electronic data capture (REDCap)–a metadata-driven methodology and workflow process for providing translational research informatics support. J Biomed Inform 42: 377–381. 18929686 10.1016/j.jbi.2008.08.010 PMC2700030

[61] Ciampa PJ, Skinner SL, Patricio SR, Rothman RL, Vermund SH, et al (2012) Comprehensive knowledge of HIV among women in rural Mozambique: development and validation of the HIV Knowledge 27 Scale. PLoS One 7: e48676. 23119087 10.1371/journal.pone.0048676 PMC3485372

[62] INSIDA (2009) National Survey on Prevalence, Behavioral Risks and Information about HIV and AIDS (2009 INSIDA). Maputo, Mozambique: Instituto Nacional de Saúde (INS), Ministry of Health (MISAU), & Instituto Nacional de Estatística (INE).

[63] Vermund SH, Blevins M, Vaz LM, Shepherd BE, Parrish DD, et al.. (2012) Poor clinical outcomes for HIV infected children on antiretroviral therapy in rural Mozambique: need for quality improvement and continuing program/community development in PEPFAR. XIX International AIDS Conference Washington, DC.

[64] Gqaleni N, Hlongwane T, Khondo C, Mbatha M, Mhlongo S, et al.. (2011) Biomedical and Traditional Healing Collaboration on HIV and AIDS in KwaZulu-Natal, South Africa Universitas Forum.

